# Physico-Chemical Properties of Calcium-Silicate vs. Resin Based Sealers—A Systematic Review and Meta-Analysis of Laboratory-Based Studies

**DOI:** 10.3390/ma15010229

**Published:** 2021-12-29

**Authors:** Viresh Chopra, Graham Davis, Aylin Baysan

**Affiliations:** 1 Department of Adult Restorative Dentistry, Oman Dental College, Department of Oral Bioengineering, Queen Mary University, Mile End Rd. Bethnal Green, London E1 4NS, UK; v.chopra@qmul.ac.uk; 2 Institute of Dentistry, Faculty of Medicine and Dentistry, Queen Mary University of London, Mile End Rd. Bethnal Green, London E1 4NS, UK; g.r.davis@qmul.ac.uk

**Keywords:** calcium silicate-based sealers, resin-based sealers, physico-chemical, meta-analysis, root canal treatment

## Abstract

Introduction: The aim of this systematic review is to analyse the effect of physico-chemical properties of calcium silicate-based sealers in comparison to epoxy resin sealers in permanent teeth using a single-cone obturation technique. Methods: The study was conducted according to the guidelines of Cochrane Handbook for Systematic Reviews of Interventions and Preferred Reporting Items for Systematic Review and Meta-Analyses (PRISMA) statement. Literature search was performed using the PubMed/MEDLINE, Cochrane Central Register of Controlled Trials, Web of Science, DOAJ, Open Gray with no language restrictions until October 2020. Two reviewers assessed the studies for eligibility. Grading of Recommendations, Assessment, Development, and Evaluations (GRADE) was carried out to assess the evidence. Meta-analysis of the pooled data with subgroups was performed using the RevMan software (*p* < 0.05). Results: Results from the 28 included studies showed that the mean difference in adaptation to root canal walls (marginal adaptation, interfacial gaps and void volume) for both sealers were non-significant. However, void volume values showed a significant mean difference (*p* < 0.00001) favouring the calcium silicate-based sealers. The pooled meta-analysis reported statistically significant differences for apical microleakage (*p* = 0.0007) whilst there were non-significant mean differences for fracture resistance (*p* = 0.09) and push-out bond strength (*p* = 0.63). The heterogeneity among the included studies was 97% (I^2^). Conclusions: Within the limitations of this review, calcium silicate-based sealers demonstrated a similar or superior performance in comparison to resin-based sealers in terms of the physico-chemical properties.

Significance: The observations from the included laboratory-based studies would be informative in the evaluation of innovative root canal sealers and provide initial evidence that prepares a substantial ground for controlled randomised clinical trials in future.

## 1. Introduction

The purpose of root canal treatment is to reduce the number of microbial entities, eliminate infection and initiate peri-radicular healing [1,2]. The success of root canal therapy depends upon the triad of instrumentation, disinfection, and three-dimensional (3D) obturation [3]. The 3D obturation is essential to prevent microleakage and avoid re-infection of the root canal system [4,5]. The most commonly used obturation material: Gutta percha (GP) [5] is unable to adhere to the dentine walls, therefore the GP alone is insufficient to achieve a desirable 3D obturation. The use of root canal sealer is required along with the GPs to achieve a fluid tight seal [6].

Resin based sealers (RBS) are considered as gold standard with regards to the physicochemical properties in comparison to other commercially available sealers to be used with gutta percha [7]. These sealers exhibit low solubility, disintegration and adequate dimensional stability [7,8]. However, the resin-based sealers fail to achieve the 3D obturation due the absence of chemical bonds between the polyisoprene of GP cones and components of sealers leading to potential microleakage [9]. Unfortunately, resin sealers lack biomimetic properties that would be essential for the long-term seal of root canal systems [10]. In this respect, bioactive calcium silicate-based sealers have been developed to overcome these challenges and provide satisfactory clinical outcomes [11,12]. According to their interaction with the host tissue, these bioactive sealers can be categorised as bioinert; non-interactive with biological systems (Alumina, zirconia), bioactive; durable tissues that can undergo interfacial interactions with surrounding tissues (bioactive glasses, bioactive glass ceramics, hydroxyapatite, calcium silicates) and biodegradable; soluble or resorbable, eventually replaced or incorporated into tissue (Tricalcium phosphate, Bioactive glasses) [13].

As a separate note, calcium silicate-based sealers (CSBs) demonstrate favourable properties such as hydrophilic nature, high pH above 12, antimicrobial properties, expansion on setting, insolubility in the presence of tissue fluids [14]. The setting reaction of their main component calcium silicate results in precipitation of calcium phosphate [13]. In addition, calcium phosphate enables to form the chemical composition and crystalline structure similar to teeth and bone apatite materials. The improved bond between a sealer and root dentine encourages bioactivity and tissue growth in comparison to other commercially available root canal sealers [12].

Single-cone obturation technique with rotary instrumentation systems has been recommended with the CSBs [15,16], since this combination minimises the pressure applied to root canal walls resulting in a uniform mass in comparison to other obturation techniques [17,18,19]. Interestingly, studies observing the effect of obturation techniques and bond strength of sealers to root canal dentine demonstrated a relatively high push-out bond strength for the CSBs compared to the RBSs [20,21,22]. However, there are contrasting results showing low bond strength values and resistance to dislodgment of CSBs in comparison to the RBSs [7,22,23,24,25,26,27]. This limited and contradictory evidence [24,25,26,27] on the physico-chemical properties of the CSBs would have an impact on the choice of sealers and outcome of root canal therapy. Therefore, the aim of this systematic review and meta-analysis was to compare the effect of physico- chemical properties of CSBs vs. RBS on the outcome of root canal treatment using a single cone obturation technique in permanent teeth.

## 2. Materials and Methods

### Literature Research

**Figure 1:**
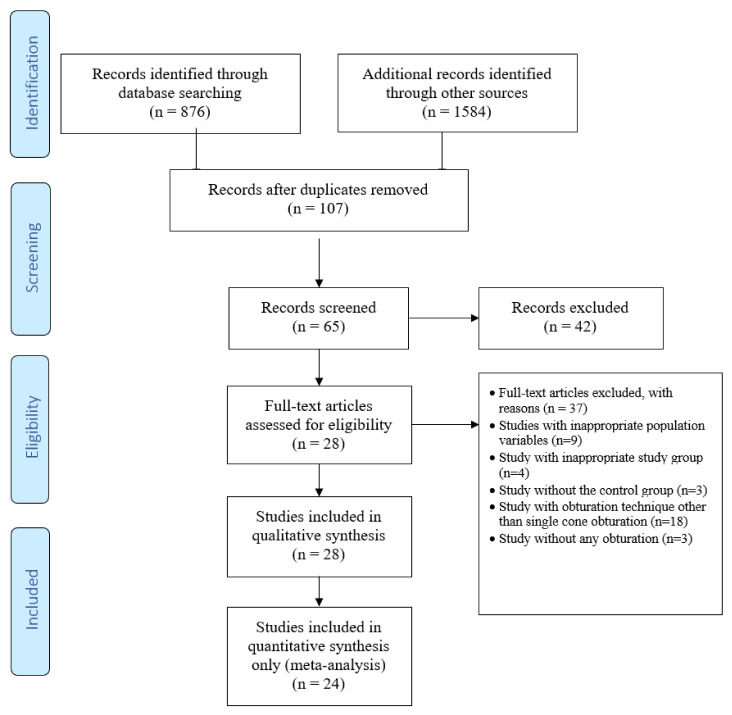
PRISMA flow diagram.

This review was conducted according to the guidelines of Cochrane Handbook for Systematic Reviews of Interventions and Preferred Reporting Items for Systematic Review and Meta-Analyses (PRISMA) statement (Figure 1). The PICOS framework (Table 1) was used to formulate the following research question ‘Is there a difference between calcium silicate-based sealers and resin-based sealers in terms of physico-chemical properties on the outcome of root canal treatment in extracted permanent teeth using a single cone obturation technique?’

## 3. Literature Screening and Study Selection

A comprehensive search was carried out in PubMed/MEDLINE, DOAJ, Cochrane Central Register of Controlled Trials and Web of Science to identify studies without any language restriction between April 2019 and October 2020. Searches in the references of the included studies (cross-referencing) were also conducted. Search on Gray literature was performed using Google, Greylit, and OpenGrey. Medical Subject Headings (MeSH) terms, keywords, and other free terms related to PICO question were used with Boolean operators (OR, AND) to combine searches. The same keywords were used for all search platforms by following the syntax rules of each database.

## 4. Data Extraction

Literature search results were de-duplicated by using EndNote X7 software (Thomson Reuters, New York, NY, USA). Studies have initially been screened based on titles, abstracts according to the scope (i.e., studies that report the physical properties of CSBs and resin based endodontic sealers), and publication types (i.e., reviews, comments, letters, or abstracts). Two reviewers (VC & AB) independently selected the included studies according to inclusion and exclusion criteria using the PICO. The full texts were then accessed. Any discrepancies among authors/reviewers were resolved after careful discussion with the third author (DG).

The data was extracted from each included study by the same reviewers for study identification number, authors, study design, sample type, method of sample preparation, storage of samples, sample size, type of sealers, variables, method of analysis, method of outcome assessment, follow-up and author’s conclusions. Following this, evidence table was created. Authors of the included studies were also approached via e-mail for any missing data.

### Quality Assessment and Risk of Bias Analysis

Grading of Recommendations, Assessment, Development, and Evaluations (GRADE) was carried out to assess the evidence. The risk of bias assessment was adapted from the previous studies [28,29,30] (Table 2). The evaluation was based on the description of the following parameters for the quality assessment of study: sample size calculation, samples with similar dimensions, randomisation process, standardisation of instrumentation and obturation procedures, endodontic treatment performed by a single operator, blinding of the observer and statistical analyses carried out. If the authors reported the parameter, the article had a Y (yes) for that specific parameter; if it was not possible to find the information, the article received an N (no). The studies that reported 1–3 items were classified as high risk of bias, 4–6 as medium risk, and 7–8 as low risk. The assessment was carried out by two reviewers, then any disagreements were resolved by discussion and followed up with a consensus.

## 5. Results

The flowchart of the systematic review is shown in Figure 1. The initial electronic database search on PubMed/MEDLINE and Cochrane library resulted in 876 titles and google scholar search resulted in 1584 titles. After removal of duplicates and screening of the abstracts, a total of 107 relevant titles were selected. A total 65 out of 107 articles were then chosen for the full-text evaluation which also included studies through hand searching of the reference lists of the selected studies. Following the inclusion and exclusion criteria using the PICO, a total of 28 studies were selected.

Nine studies with inappropriate population variables, four studies with inappropriate study group, three studies without the control group whilst 18 studies due to different obturation techniques other than the single cone obturation and three studies due to the lack of information on obturation techniques were excluded.

Subsequently, four out of 28 studies were excluded, as two studies [31,32] used different comparative variables and two studies [33,34] failed to standardise their assessment criteria. Meta-analysis could only be performed for 24 studies due to the discrepancies in distribution of samples, lack of assessment criteria standardisation and differences in comparative variables in the excluded studies.

### 5.1. Physico-Chemical Properties and Meta-Analysis

The main characteristics of the included studies are shown in Table 3. Ten studies on the adaptation to root canal walls were included [30,31,32,35,36,37,38,39,40,41]. In comparison to the RBS, CSBs showed similar values of void volume marginal adaptation in five studies [32,36,37,38,39] whilst at the apical, mid, and coronal thirds, these values were high in three studies [31,35,41] and low in one study [38].

#### 5.1.1. Marginal Adaptation

**Figure 2:**
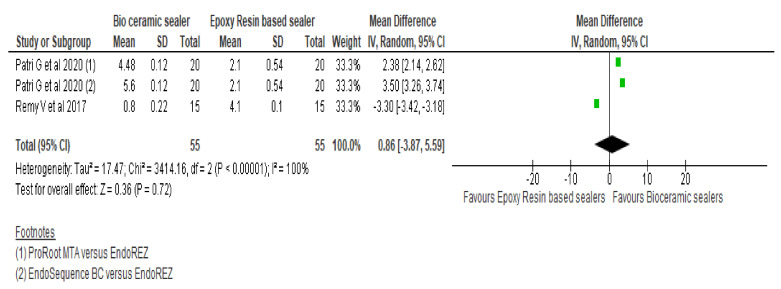
Forest plot comparing bioceramic and resin-based sealers for adaptability to the root canal walls with regards to adaptability at the apical third of root canal system.

**Figure 3:**
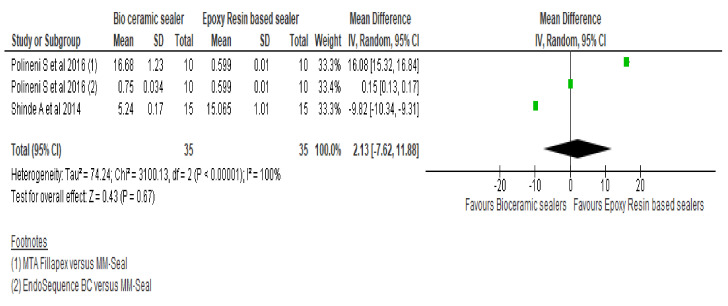
Forest plot comparing bioceramic and epoxy resin-based sealers for adaptability to the root canal wall with respect to interfacial gaps at the apical third of root canal system.

Forest plot of the pooled comparison (Figure 2) between RBS and CSBs for marginal adaptation demonstrates mean difference of −0.86 [95% CI −3.87, 5.59] favouring RBS [95% CI −3.87, 5.59] with high heterogeneity (Q = 3414.16, heterogeneity *p* < 0.00001, I^2^ = 100%). There were no significant differences between the two groups. However, forest plot of the pooled comparison between CSBs (n = 66) and RBS (n = 66) for interfacial gaps (Figure 3) shows statistically significant standardised mean void volume values (*p* < 0.00001) by favouring the CSBs with a mean difference and confidence interval of −0.90 [−1.30, −0.51] with heterogeneity of 14% (I^2^).

Subgroup analysis according to the root third [30,51] showed significantly high standardised mean void volume values for coronal third (*p* = 0.0007), middle third (*p* = 0.10) and apical third (*p* = 0.08) respectively favouring CSBs.

#### 5.1.2. Fracture Resistance

**Figure 4:**
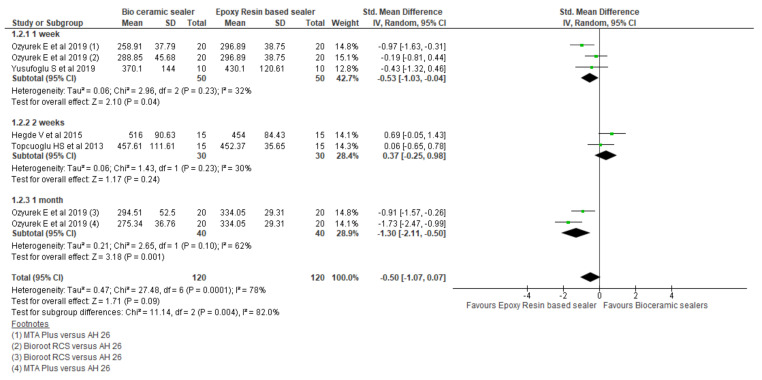
Forest plot of the pooled analysis and the subgroup analysis comparing bioceramic sealers and epoxy resin-based sealers for fracture resistance.

Four studies on fracture resistance were included [36,37,54,56] with values (FRV) mentioned in Newton unit and time intervals ranged from one week to a month. CSBs demonstrated similar FRVs when compated to RBS in three studies [36,37,56], whilst had superior values in one study [54]. Figure 4 exhibits the forest plot of the pooled comparison between the CSBs (n = 120) and RBS (n = 120) demonstrating non-significant standardised mean FRVs (*p* = 0.09) favouring the RBS. The mean difference and confidence intervals were −0.50 [−2.11, −0.50] with heterogeneity of 14% (I^2^).

According to the time interval, one-week [36,37] and one-month intervals favoured the RBS with statistically significant standardised mean of FRVs (*p* = 0.04), (*p* = 0.001) respectively whilst two-week interval [54,56] favoured the CSBs group with the mean difference value of 0.37 [−0.25, 0.98], (*p* = 0.24). The heterogeneity was 30% (I^2^). 

#### 5.1.3. Pushout Bond Strength

**Figure 5:**
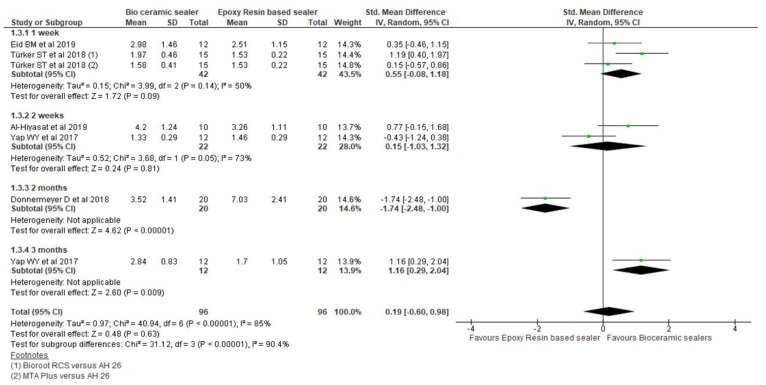
Forest plot of the pooled analysis and the subgroup analysis comparing bioceramic and epoxy resin-based sealers for push out bond strength.

Six studies assessing the pushout bond strength (PBS) in MPa were included [13,34,38,44,49,52]. Both the targeted sealer groups showed similar PBS in one study [49]. Four studies [13,34,44,52] demonstrated high PBS with the CSBs in comparison to the RBS. However RBS showed superior results when compared to the BCS in one study [38]. Figure 5 exhibits the forest plot of the pooled comparison between the CSBs (n = 96) and RBS (n = 96) groups at varying time periods. There was non-significant standardised mean of PBS (*p* = 0.63) favouring the bioceramic sealers. 

In addition, there were two studies with one-week [34,44] (*p* = 0.09) and two-week intervals [13,49] favoring the CSBs (*p* = 0.81). However, the two-month interval [38] demonstrated statistically significant values for the RBS (*p* < 0.00001). Interestingly, there were statistically significant values favoring the CSBs (*p* = 0.009) which was 1.16 MPa [0.29, 2.04] at the three-month interval [49]. 

#### 5.1.4. Penetration Depth

Three studies assessing penetration depth values in millimetres (mm) and micro-meters (µm) using Confocal Laser Scanning Microscopy (CLSM) were included [35,44,46]. The CSBs showed a better penetration depth in one study [44], whilst there were no significant differences in two studies [35,46]. In addition, one study concluded that the RBS had a greater penetration buccolingually in teeth with the butterfly effect when the root canal system have high density of dentinal tubules in the bucco-lingual direction [51].

#### 5.1.5. Apical Microleakage

Solubility is the mass loss of a material during a period of immersion in water. According to ANSI/ADA Specification 57 [34], the solubility of a root canal sealer should not exceed 3% by mass. A highly soluble root canal sealer would permit the formation of gaps within and between the material and root dentine causing leakage at the interfaces.

**Figure 6:**
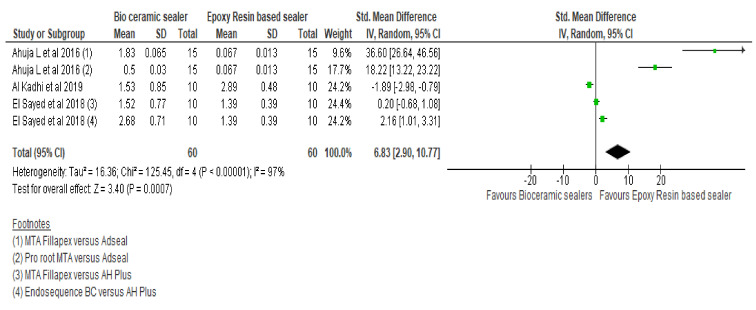
Forest plot comparing bioceramic and epoxy resin-based sealers for apical microleakage.

Four studies were included for the assessment of apical microleakage [32,40,43,50] in mm with a total of 80 in the CSBs whilst 55 teeth in the RBS groups. Figure 6 demonstrates the forest plot using the random effect model showing standardised mean difference of 6.83 mm favouring the RBS [95% CI 2.90, 10.77] as well as showing statistically significant difference (*p* = 0.0007) between these groups. The heterogeneity of the included studies in the meta-analysis was high (Q = 125.45, heterogeneity *p* < 0.00001, I^2^ = 97%). 

#### 5.1.6. Coronal Discoloration

Only one study was included for evaluation of coronal discoloration at 1 and 3-month intervals. At all assessment times, there were no significant differences between CSBs and RBS sealers [33]. However, the authors concluded that all sealers caused discolouration that increased over time. The CSBs demonstrated superior colour change in comparison to the RBS group. The reported tooth discolouration depended on the chemical composition of the sealer rather than the type of sealant with a small sample in each group (n = 10). The authors also failed to report the process used power calculation of the samples.

#### 5.1.7. Bacterial Leakage

One study using a total of 80 extracted mandibular and maxillary canines was included. The authors evaluated the sealing ability of RBS and CSBs for a period of 60 days using the bacterial leakage model with *Enterococcus faecalis*. The RBS showed low number of leaked samples as compared to the CSBs, however the mean values of apical leakage was statistically similar. It was therefore concluded that the sealing ability of the CSBs was equivalent to the RBS when used with a single-cone obturation [53].

## 6. Discussion

This systemic review with the meta-analysis [56] was the first to assess the physico-chemical properties of calcium silicate vs. resin-based sealers. The adaptation to root canal walls was evaluated using the marginal adaptation, interfacial gaps and void volume in cubic millimetres with mean percentages. The marginal adaptation [31,48] and interfacial gaps [39,53,55] were assessed using the Scanning Electron Microscope whereas void volume [30,41,42,47,51] was assessed by Micro-computed Tomography. Kim et al. [30] stated that the voids in the root canal obturation might weaken the filling quality and serve as a hub for microbes by transporting contaminants along the root canal system. 

Various studies reported that RBS could penetrate deep into the dentinal tubules due to the flowability, long setting time, and provide long-term dimensional stability [48,53,57,58]. Germain et al. [41] suggested that EndoSequence (CSBs) reduced film thickness, improved flow and hardening on contact with moisture within the root dentine. This resulted in the optimum marginal adaptation followed by the ProRoot MTA sealer (CSBs) [59]. Fewer interfacial gaps in the CSBs as compared to the RBS [39] could be attributed to the alkaline caustic effect of the products formed by calcium silicate sealer hydration reaction. This would degrade the collagenous component of the interfacial dentine facilitating the penetration of sealers into the dentinal tubules [39,59]. In addition, McMichael et al. [60] concluded that fine particle sizes and premixed consistency with a capillary tip introductory system might have enhanced the CSBs penetration to the full length of root canal systems.

The CSBs demonstrated less and statistically significant mean percentage values (mm^3^) when compared to RBS with regards to void volume [31,41,47,51]. The degree of adhesion of the sealer to the dentine walls depends on the intermolecular surface energy, cleanliness of the dentine, surface tension and wetting ability of the sealer [42]. Huang et al. (2017) demonstrated that all sealers had less void volume in the apical third (n = 10 for each group) [47]. This might be due to more pressure during the root canal preparation, irrespective of the obturation techniques. It is important to avoid void formation in sealers especially in cases where the sealers and core material would shrink/degrade and where obturation relies more on sealers than core materials i.e., single cone technique [47]. However, all the studies evaluated the void volume at short time intervals (5–10 days). Further investigation for potential changes with time is required and their degree of clinical relevance is yet to be authenticated. The effect of all parameters with regards to adaptation should also be assessed in future studies.

Endodontically treated teeth are weaker and more prone to fracture than vital teeth [56,57]. Topçuoğlu et al. [56] suggested that adhesion of sealers to the surfaces of root dentine might strengthen the remaining tooth structure, thereby contributing to the long-term success. However, there was lack of information on the standardisation of parameters such as root canal preparation, obturation technique used, storage conditions of test teeth during the study. Hegde et al. [54] and Cobankara et al. [61] demonstrated higher FRV with CSBs (EndoSequence BC) in comparison to the RBS (AH Plus) which might be due to the presence of sealer’s nanoparticles resulting in chemical bonding and deep penetration of the sealer into canal irregularities and dentinal tubules. However, Ozyurek et al. [36] reported least FRV of MTA Plus sealer (*p* < 0.05) as this material showed high porosity, water solubility, and water sorption resulting in its lower fracture resistance values. In all included studies, both CSBs and RBS were able to increase the force to fracture in single-rooted endodontically treated teeth. However, none of the studies mentioned the intervals between the extraction and testing times. In addition, the FRV of multi-rooted teeth were not assessed. 

Single cone technique has been recommended for obturation with the CSBs [13,28]. Sealer contains nanoparticles that facilitates sealer penetration into the canal irregularities and dentinal tubules, therefore providing an optimum interface between the root canal walls and sealer. However, none of the studies reported the application process of these sealers to the root canals. The PBS values for the CSBs were similar or high when compared to the RBS except in the study conducted by Donnermeyer et al. [38]. Almeida et al. [62] suggested that the adequate performance of the target sealers might be related to the formation a chemical bond to dentine by the production of hydroxyapatite during setting. The sealer penetration depth was evaluated under the CLSM as this system allows the detection of sealer penetration along the canal circumference of each sample using the fluorescence. With the CLSM, the formation of high contrast points shows both, the sealer penetration within the dentinal tubules and the sealer within the segments at various depths [35,63]. El Hachem et al. [35] compared the penetration depth of RBS and CSBs at 1 and 5 mm with the single cone obturation in 96 human central incisors. There were no significant differences between these groups at 1 mm, however maximum and mean penetration depths were significantly low at 5 mm for RBS in comparison to CSBs (*p* = 0.012). The authors concluded that calcium silicate-based sealer could penetrate dentinal tubules without applying the intra-canal compaction pressure by the employment of the single-cone technique. However, these authors failed to report the effect of smear layer on the penetration depth. It should be noted that this finding would be clinically significant, as it would remove the necessity to use excessive force during obturation procedures, therefore avoiding the generation of root canal cracks or fractures [46]. In this respect, Türker et al. [44] showed significantly high penetration depths for the MTA Plus compared to the BioRoot RCS and AH 26 sealers when the smear layer was preserved. However, the BioRoot RCS showed the low penetration depth in the absence of smear layer.

Investigating the solubility of CSBs is a major area for debate. In this respect, the superior or comparative sealing ability of the CSBs was reported [52,53], however Ahuja et al. [50] demonstrated significantly high apical microleakage values for the CSBs resulting in a high standardised mean difference which favours the RBS. In addition, Al-Haddad et al. [63] reported that RBS are slightly acidic and might result in self-etching when in contact with dentine, thereby enhancing interfacial bonding and adaptation with a reduction in the apical microleakage. Interestingly, Al-Kadhi et al. [32] showed superior performance with the CSBs. However, their tabulation and reconfirmation of the results were unclear and sample size for each group was small (*n* = 10). As a separate note, El Sayed et al. [40] reported the lowest apical leakage value with the CSBs, however there was no significant difference when compared with the RBS. High solubility of CSBs might raise questions on the long-term sealing ability [62]. Properties such as solubility and exchange of ions are responsible for specific interactions between CSBs and the dentine walls (mineral infiltration zone) [63,64]. Solubility of a sealer might also be overestimated due to the chemistry of CSBs. This would explain the differences between the high and low solubility values in various studies [58]. However, the contradictory results regarding the solubility of CSBs might be due to their hydrophilic nature as the fluid environments (use of culture media) are known to influence solubility results [54].

Alotaibi et al. [33] reported the CSBs (Totalfill) showing the highest whilst the RBS (AH Plus) presented the lowest colour changes both at one and three months, however these differences were not statistically significant. These authors observed the colour change on the crown as a whole, however any specific areas on the surface of the crown were not mentioned. In addition, the small sample size to observe colour change affected the quality of this study.

Ethical approval increases the legitimacy of research findings and is important for making decisions based on the research results. Eleven included studies [30,35,37,39,42,43,44,45,46,63] mentioned the ethical approval process and four studies included [42,47,49,63] the information related to international funding and collaborations whilst the remainder were self-funded.

The present systematic review and meta-analysis focused on specific physico-chemical properties such as apical microleakage, solubility, discoloration, pushout bond strength, penetration depth, fracture resistance and marginal adaptation. However, importance of other properties; setting time, microhardness, wettability, flowability and radiopacity in root canal treatment needs to be considered in future studies. It should be noted that the data substrated from the included study in the present review has been homogenous and the physicochemical properties was restricted to few properties i.e., marginal adaptation, fracture resistance, pushout bond strength. Therefore, the results from the included studies showed a multivariate effect rather than univariate with a non-linear regression.

The overall results of this meta-analysis showed that the CSBs presented an optimum performance with similar or better results when compared to the RBSs with respect to the physico-chemical properties except for apical microleakage. However, direct extrapolation of the findings from the included studies might not completely represent clinical situations, however these results could be useful in the evaluation of bioceramic sealers and provide evidence that might prepare the ground for controlled randomised clinical trials [7]. In addition, future laboratory-based studies could consider using teeth with similar dimensions as comparing teeth with different dimensions or a single rooted with multirooted teeth can cause a discrepancies in observations. The power analysis for sample size would provide an appropriate number, since most of these studies presented with small sample sizes. The study period of the included studies varied from 24 h to one month which might have affected the results, since the behaviour of the material continues to change over a period of time. Therefore, further studies could be conducted by defining time points with extended study duration to have a better correlation with clinical setup. To avoid inter-operator variability, each procedure including root canal preparation, irrigation, and obturation should be performed by one operator. With respect to performance and detection bias, both the operator and observer should be blinded to the groups. To promote quality and transparency in reported results of laboratory-based studies of endodontically treated teeth, standardisations of root canal treatment and assessment of outcome procedures are also crucial [7,28,29,64].

## 7. Conclusions

The physical properties of root canal sealers have a major impact on the quality of 3D-obturation. Due to the hydrophilic environment of root canal systems, water resorption and solubility of root canal sealers are important factors for the stability of obturations. Minimal microleakage of the sealer and high push-out bond strength are needed to endure the dynamic tooth environment.

Within the limitations of this review, calcium silicate sealers showed satisfactory performance with similar or better results when compared to the resin-based sealers in terms of Physico-chemical properties.

## Figures and Tables

**Table 1:** The search strategy and PICOS tool.

Search Strategy	
Focused Question	Is there a difference between calcium silicate based sealers and resin-based sealers in terms of physico-chemical properties on the outcome of root canal treatment using a single cone obturation technique for extracted permanent teeth?
PICO Strategy	
Population	(Permanent Dentition [MeSH] OR Adult Dentition OR Secondary Dentition OR Permanent teeth OR Teeth OR Extracted teeth OR Root Canal Obturation [MeSH] OR Single cone obturation
Intervention (#1)	(Bioceramic sealer OR Endosquence BC OR iRoot Plus OR MTA fillapex OR Totalfill BC OR tricalcium phosphate OR tricalcium phosphate ceramic sealer OR Calcium silicate sealer OR Calcium phosphate sealer OR Endodontic sealer OR Root canal sealer
Comparisons (#2)	(Epoxy resin-based root canal sealer OR AH Plus OR AH 26
Outcomes (#3)	(Depth of penetration OR Adaptability OR Void volume OR Seal ability OR Adhesiveness OR Tooth discoloration OR Fracture resistance OR Fracture strength OR Bond strength OR Push-out bond strength OR Root fracture OR Anti-microbial OR Penetration
Study design (#4)	(In Vitro Techniques [MeSH] OR In vitro studies OR In vitro studies OR Ex vivo studies
Search Combination	#1 AND #2 AND #3 AND #4
Database Search	
Language	No restriction (Articles in English language or other language where English translation is possible.)
Electronic Databases	PubMed/MEDLINE, Cochrane Central Register of Controlled Trials, Web of Science
Journals	Journal of Endodontics, International Endodontic Journal, Australian Endodontic Journal, Clinical Oral Investigations, Journal of Conservative Dentistry, Journal of American Dental Association. Brazilian dental journal, Journal of physics, Materials, Dental materials etc.
Period of Publication	1 January 2011 to 31 August 2020

**Table 2:** Risk of bias assessment of included studies.

Sr. No	Study Id	Sample Size Calculation	Samples with Similar Dimensions	Teeth Randomization	Standardization of Instrumentation Procedures	Standardization of Filling Procedures	Endodontic Treatment Performed by a Single Operator	Blinding of the Observer	Statistical Analysis Carried Out	Risk of Bias
1.	Kim J et al. (2020) [30]	No	Yes	Yes	Yes	Yes	No	No	Yes	Medium risk
2.	Patri G et al. (2020) [31]	No	Yes	Yes	Yes	Yes	No	Yes	Yes	Medium risk
3.	Al-Hiyasat et al. (2019) [13]	No	Yes	Yes	Yes	Yes	No	No	Yes	Medium risk
4.	Al-Kadhi AM et al. (2019) [32]	No	Yes	Yes	Yes	Yes	No	No	Yes	Medium risk
5.	Alotaibi RM et al. (2019) [33]	No	Yes	Yes	Yes	Yes	Yes	No	Yes	Medium risk
6.	Eid BM et al. (2019) [34]	No	Yes	Yes	Yes	Yes	No	No	Yes	Medium risk
7.	El Hachem R et al. (2019) [35]	No	Yes	Yes	Yes	Yes	Yes	No	Yes	Medium risk
8.	Ozyurek E et al. (2019) [36]	No	Yes	Yes	Yes	Yes	Yes	No	Yes	Medium risk
9.	Yusufoglu S et al. (2019) [37]	No	Yes	Yes	Yes	Yes	Yes	No	Yes	Medium risk
10.	Donnermeyer D et al. (2018) [38]	Yes	Yes	Yes	Yes	Yes	No	No	Yes	Medium risk
11.	Eltair M et al. (2018) [39]	No	Yes	Yes	Yes	Yes	Yes	Yes	Yes	Low risk
12.	El Sayed et al. (2018) [40]	No	Yes	Yes	Yes	Yes	Yes	Yes	Yes	Low risk
13.	Germain S et al. (2018) [41]	No	Yes	Yes	Yes	Yes	No	No	Yes	Medium risk
14.	Huang Y et al. (2018) [42]	No	Yes	Yes	Yes	Yes	No	No	Yes	Medium risk
15.	Salem AS et al. (2018) [43]	Yes	Yes	Yes	Yes	Yes	No	No	Yes	Medium risk
16.	Türker ST et al. (2018) [44]	No	Yes	Yes	Yes	Yes	No	No	Yes	Medium risk
17.	Yanpiset K et al. (2018) [45]	No	Yes	Yes	Yes	Yes	No	No	Yes	Medium risk
18.	Russell A et al. (2018) [46]	Yes	Yes	Yes	Yes	Yes	Yes	Yes	Yes	Low risk
19.	Huang Y et al. (2017) [47]	Yes	Yes	Yes	Yes	Yes	Yes	Yes	Yes	Low risk
20.	Remy V et al. (2017) [48]	Yes	Yes	No	Yes	Yes	No	No	Yes	Medium risk
21.	Yap WY et al. (2017) [49]	Yes	Yes	Yes	Yes	Yes	No	No	Yes	Medium risk
22.	Ahuja L et al. (2016) [50]	No	Yes	Yes	Yes	Yes	No	No	Yes	Medium risk
23.	Celikten B et al. (2016) [51]	Yes	Yes	Yes	Yes	Yes	Yes	Yes	Yes	Low risk
24.	Madhuri GV et al. (2016) [52]	No	Yes	No	Yes	Yes	No	No	Yes	Medium risk
25.	Polineni S et al. (2016) [53]	No	Yes	Yes	Yes	Yes	No	Yes	Yes	Medium risk
26.	Hegde V et al. (2015) [54]	No	Yes	Yes	Yes	Yes	No	No	Yes	Medium risk
27.	Shinde A et al. (2014) [55]	No	Yes	Yes	Yes	Yes	No	No	Yes	Medium risk
28.	Topcuoglu HS et al. (2013) [56]	No	Yes	Yes	Yes	Yes	No	No	Yes	Medium risk

**Table 3:** Articles Included in the Systematic Review: Physical-Chemical and Anti-microbial Properties of Bioceramic and Epoxy resin based Endodontic Sealers.

Property	Author, Year	Method	Material	Author’s Conclusion
Adaptation to root canal wall	Kim J. et al. (2020)	Void percentage	EndosealMTA AH Plus Jet	Endoseal MTA does not seem to reduce the voids over time when it was used with a single gutta-percha cone technique.
	Patri G. (2020)	Sealing potential and marginal adaptation	EndoSequence BC ProRoot MTA EndoREZ	Significant and better sealing ability and marginal adaptation was demonstrated by EndoSequence BC (bioceramic sealer) when compared to ProRoot MTA sealer (MTA-based sealer) and EndoREZ sealer (resin-based sealer).
	Eltair M. et al. (2018)	Areas and interfacial gaps between sealer and dentine	TotalFill BC AH Plus	All tested root canal fillings exhibited minor interfacial gaps. The BC sealer showed better adaptability than the AH Plus sealer.
	Germain S. et al. (2018)	Voids volume in the apical third	TotalFill bioceramic (NB) sealers AHPlus	Bioceramic (BC) sealers showed good all-round performance demonstrating good adaptability, and reduced voids while maintaining similar characteristics when compared with conventional resin sealer.
	Huang Y. et al. (2018)	Total ROI volume (mm^3^), object volume (dentin volume, mm^3^), volume of closed pores (mm^3^), surface of closed pores (mm^2^), volume of open pores (mm^3^), and open porosity (%)	EndoSequence BC AHPlus	By using the single cone technique, neither endoSequence or AH Plus provides a porosity-free root canal filling. The EndoSequence BC sealer may have similar sealing abilities regarding the whole root canal as the AH Plus sealer. A better sealing effect could be obtained in the coronal and middle sections of a root canal than the apical part by using the tested sealers.
	Huang Y. et al. (2017)	Void volume in (mm^3^)	Sure Seal Root Total BC Sealer AH Plus	A high incidence rate of voids was found within each sealer material and no significant difference was found among the root filling sealers.
	Remy V. et al. (2017)	Marginal adaptation	MTA Fillapex AH Plus	AH Plus sealer shows a good marginal adaptation.
	Celikten B. et al. (2016)	Voids in 3D volumes	EndoSequence BC AH Plus	All root canal sealers tested resulted in voids. The bioceramic sealers (Endo Sequence BCSealer, Smartpastebio) produced similar voids which had the fewest in the apical third of root canals.
	Polineni S. et al. (2016)	Maximum gap width (nm)	MTA Fillapex EndoSequence BC MM-Seal	Epoxy resin-based MM-Seal showedgood marginal adaptation than the MTA Fillapex. apical halves showed poor adaptation regardless of the material used than the coronal halves
	Shinde A. et al. (2014)	Mean distance from the radicular dentin to the root canal fillings was in (mm)	Endo- Sequence BC AH Plus	Endosequence BC endodontic sealers showed better adaptation to the radicular dentin as compared to AH Plus sealer.
Fracture Resistance	Ozyurek E. et al. (2019)	Fracture resistance values (FRV) in Newtons	MTA Plus BioRoot RCS AH 26	Root canal preparation lowered the fracture resistance values. All sealers increased the force values needed to fracture the filled samples compared to unfilled ones but the time factor had no effect on the reinforcement effect of root canal sealers.
	Yusufoglu S. et al. (2019)	Push-out bond strength	BioRoot RCS AH Plus	All the three root canal sealers examined in this study strengthened the prepared root canals with increased fracture resistance
	Hegde V. et al. (2015)	Forces in Newton	EndoSequence BC AH Plus	Hydrophilic obturations have shown to reinforce the strength of the root canal after instrumentation, and thus increasing the fracture resistance of the root to the stresses encountered
	Topcuoglu H.S. et al. (2013)	Forces in newtons	EndoSequence BC AH Plus	Endosequence BC sealer and AH Plus Jet were able to increase the force to fracture in single-rooted endodontically treated premolar teeth.
Bond strength	Al-Hiyasat et al. (2019)	Push-out bond strength	TotalFill AH plus	Overall the push-out bond strength of TotalFill BC sealer was significantly higher than that of AH plus sealer.
	Eid B.M. et al. (2019)	Push-out bond strength	Totalfill bioceramic Adseal sealer	The push-out bond strength of the tested TotalFill root canal sealer was higher than the pushout bond strength of Adseal resin sealer
	Donnermeyer D. et al. (2018)	Push-out bond strength	Total Fill BC AH Plus	The push-out bond strength of the investigated calcium silicate-based sealers was lower than of AH Plus. Total Fill BC showed the highest push-out bond strength of the calcium silicate-based sealers.
	Türker S.T. et al. (2018)	Push-out bond strength	BioRoot RCSMTA Plus AH 26	Dentinal tubule penetration had limited effect on the push-out bond strength of the root canal sealers.
	Yap W.Y. et al. (2017)	Push-out bond strength	TotalFill BC AH Plus	TotalFill BC TM sealer (G3) showed comparable bond strengths to AH Plus. The bond strength also exhibited an increase over a 3-month post-obturation period.
	Madhuri G.V. et al. (2016)	Push-out bond strength	Bioceramic Sealer Epoxy resin-based sealer	Endosequence BC (Bioceramic Sealer) showed the highest push-out bond strength among all the four groups. MM (Epoxy resin based sealer) showed the second highest bond strength followed by Hybrid seal (Dual cure resin based sealer)
Penetration depth	El Hachem R. et al. (2019)	Dentinal penetration depth	BC Sealer AH Plus	BC Sealer and NTS demonstrated better dentinal tubule penetration results compared to AH Plus.
	Türker S.T. et al. (2018)	Dentinal penetration depth	BioRoot RCSMTA Plus AH 26	Dentinal tubule penetration had limited effect on the push-out bond strength of the root canal sealers.
	Russell A. et al. (2017)	Dentinal penetration depth	MTA Fillapex AH Plus	Coronal sections of roots have superior adaptation and penetration compared with middle sections. Penetration in middle sections was significantly more favourable in teeth without the butterfly effect.
Apical Microleakage	Al-Kadhi et al. (2019)	Apical linear dye penetration	Total fill BC Acroseal	No sealer can completely prevent microleakage, but the bioceramic is superior in performance to the other Sealers
	El Sayed et al. (2018)	Apical linear dye penetration	MTA FillapexEndoSequence BC AH Plus	Higher apical leakage values were observed with single-cone gutta-percha/EndoSequence BC as compared gutta-percha/AH Plus, single-cone gutta-percha/MTA Fillapex
	Salem A.S. et al. (2018)	Apical linear dye penetration	Total fill BC AH Plus	Total Fill BC was equivalent to AH Plus in apical sealing ability when using single cone.
	Ahuja L. et al. (2016)	Apical linear dye penetration	MTA Fillapex Pro RootMTA Adseal sealer	Adseal sealer was better in providing the apical seal than Proroot MTA and MTA Fillapex
Coronal discoloration	Alotaibi R.M. et al. (2019)	Coronal color change	TotalFill AH Plus	All sealers tested result in a measurable and gradual tooth color change. While the bioceramic sealer resulted in a slightly higher color change compared to calcium hydroxide- and resin-based sealers, the difference was not considerable.
Apical Bacterial leakage	Yanpiset K. et al. (2018)	Bacterial leakage test with E. faecalis	Bioceramic sealer AH Plus	In roundly-prepared canals, the epoxy resin sealer had lower amount of leaked samples as compared to bioceramic sealers using single cone gutta percha for bacterial leakage at 60 days.

## Data Availability

The data presented in this study are available on request from the corresponding author.
